# Understanding and Measuring Help-Seeking Barriers among Intimate Partner Violence Survivors: Mixed-Methods Validation Study of the Icelandic Barriers to Help-Seeking for Trauma (BHS-TR) Scale

**DOI:** 10.3390/ijerph19010104

**Published:** 2021-12-23

**Authors:** Karen Birna Thorvaldsdottir, Sigridur Halldorsdottir, Denise M. Saint Arnault

**Affiliations:** 1 School of Health Sciences, University of Akureyri, 600 Akureyri, Iceland; sigridur@unak.is; 2 Department of Health Behavior and Biological Sciences, University of Michigan, Ann Arbor, MI 48109, USA; starnaul@umich.edu

**Keywords:** interpersonal trauma, intimate partner violence, help-seeking, barriers, mental health, trauma recovery, survivor-centered, cross-cultural adaptation, validation, mixed-methods research

## Abstract

Intimate partner violence (IPV) against women is a global human rights violation of vast proportions and a severe public health problem. Despite high rates of adverse outcomes related to IPV, help-seeking and service utilization among survivors is low. This exploratory sequential mixed-methods study using a combined etic–emic approach describes the validation of the Icelandic Barriers to Help-Seeking for Trauma (BHS-TR) scale. The qualitative phase involved developing new items based on the experiences of 17 Icelandic IPV survivors, identifying barriers including beliefs that help-seeking is a sign of weakness, and the desire to safeguard oneself from re-traumatization. The quantitative phase examined the psychometrics of the BHS-TR in a sample of 137 IPV survivors in Iceland. Results supported an eight-factor structure (Financial Concerns; Unavailable/Not Helpful; External Constraints; Inconvenience; Weakness/Vulnerability; Problem Management Beliefs; Frozen/Confused; and Shame), which when grouped comprised two indices of Structural and Internal Barriers. The scale’s internal consistency was high (α  =  0.87), and the results provided evidence of convergent, discriminant, and known-group validity. This study adds to the growing literature supporting the advantages of applying mixed methods for instrument development and validation, and its results highlight the significance of giving rise to the voices of survivors. The BHS-TR is the first trauma-specific and survivor-centered measure of help-seeking barriers available in Iceland. It can be used to provide valuable information that may guide the development of evidence-based interventions to break down barriers and help survivors find ways to trauma recovery.

## 1. Introduction

### 1.1. Intimate Partner Violence against Women

Violence against women is a global human rights violation of vast proportions and a severe public health problem [1,2]. A landmark multi-country study led by the World Health Organization (WHO) showed that women are more at risk of experiencing violence in intimate relationships than anywhere else [3]. While all genders may experience intimate partner violence (IPV), most survivors self-identify as women. IPV is deeply rooted in gender inequality and harmful cultural norms [2,3]. To date, it remains the most widespread form of violence against women, making it the most common form of gender-based violence (GBV) globally [2,4]. Estimates from WHO indicate that the lifetime prevalence of IPV ranges from 16% to 37%, depending on the region [4].

IPV is generally a traumatic experience, and the high prevalence of acute and chronic health problems are well documented, including physical injuries, somatic symptoms, post-traumatic stress disorder (PTSD), depression, and suicide ideation [5,6,7,8]. Despite the high rates of adverse health outcomes, studies have shown that help-seeking among IPV survivors is low. Many survivors never disclose the violence to anyone, and those who do most often choose support from friends and family and are substantially less likely to seek help from formal sources, such as healthcare and social services, even despite their perceived need for such support [3,9,10,11]. These findings may reflect the limited availability or accessibility of services for abused women in some countries. However, low rates of formal help-seeking are also common in countries that are relatively well supplied with resources for survivors [3,12,13], the Nordic Region included [14,15,16].

### 1.2. Barriers to Help-Seeking

The majority of the IPV help-seeking literature to date focuses on leaving an abusive relationship. While these studies are critical, it is essential to move beyond the often first steps of the help-seeking journey, to an increased focus on the trauma recovery trajectory [17,18,19]. The existing research has shed light on several levels of help-seeking barriers among IPV survivors, including sociocultural, structural, interpersonal, and individual levels [20,21,22,23]. Furthermore, studies have indicated that survivors with depression, PTSD, and low sense of coherence face even more significant barriers as symptom burden and seeing the world as unpredictable and meaningless can make it more challenging to take action [24,25,26]. The burden of mental illness stigma, e.g., concerns for negative stereotyping, status loss, and discrimination, is also a significant deterrent to help-seeking and service utilization [27,28].

### 1.3. Existing Measures

In a recent scoping review [29] on mental health related-measures, 35 help-seeking measures were identified, of which only 10 were validated. Most of the measures focused on attitudes towards help-seeking or intentions to seek help. The Attitudes Toward Seeking Professional Psychological Help Scale (ATSPPH) [30] was the most widely used measure, and few assessed actual help-seeking behaviors. In another review, help-seeking measures were classified into similar attitudes and intentions categories but added a specific barriers category that included five measures [31]. A strength of barrier-based measures is that they are more tied to behavior than attitudinal measures [32]. Nonetheless, most of the existing measures solely focus on barriers to formal sources of help, primarily mental health treatment, and very few are trauma-specific.

### 1.4. The Barriers to Help-Seeking for Trauma Scale

The Barriers to Help-Seeking for Trauma (BHS-TR) scale was developed from an existing mental health barriers measure, the Barriers to Seeking Care scale [33], and adapted for use with GBV survivors. The BHS-TR included the 25 items from the original measure plus an additional nine trauma-specific and survivor-centered items (34-item version). The early work on the scale indicated that the barriers could be grouped into structural and internal dimensions [26], which was later confirmed in a psychometric study among American GBV survivors [34]. Moreover, a seven-factor structure was revealed, creating a 24-item version of the English BHS-TR, which was found to be reliable and valid.

The BHS-TR was translated and cross-culturally adapted into the Icelandic language and context [35], creating the first Icelandic measure on help-seeking barriers after trauma. In order to capture all possible nuances, the full 34-item version was used in the effort to develop the Icelandic BHS-TR. The scale was pretested with Icelandic IPV survivors, which provided preliminary evidence of relevance and content validity. However, most survivors in that study also mentioned barriers that they thought were missing from the scale. This finding demonstrated the need to further evaluate and adapt the Icelandic BHS-TR to better capture the reality and experiences of survivors seeking help in Iceland.

### 1.5. Study Aims

The aims of this mixed-methods validation study were to: (1) develop new barrier items for the Icelandic BHS-TR scale based on the experiences of IPV survivors in Iceland and pretest those items through cognitive interviewing, (2) evaluate the construct validity of the Icelandic version of the scale by examining its factor structure, dimensionality, convergent validity, discriminant validity, and known-group validity, and (3) evaluate the reliability by examining the internal consistency of the scale.

## 2. Materials and Methods

### 2.1. Study Design

This study was conducted following well-established guidelines and best practices for adapting and validating instruments [36,37]. A combined etic (universal) and emic (culture-specific) strategy using mixed methods was employed as recommended to enhance the construct validity of instruments for use outside their culture of origin [38,39]. We used a mixed-method exploratory sequential design [40], conducted in two phases with equal emphasis given to both phases. In this design, the Qualitative (Qual) phase occurs first, both the data collection and analysis, followed by the Quantitative (Quan) phase. This type of integration at the methods level is called building. It occurs when the findings from one data collection procedure inform the data collection approach of the subsequent procedure [40,41]. We implemented four legitimation strategies to ensure the highest design quality: sample integration, inside-outside, weakness minimization, and multiple validities [42].

The Qual phase of this study included three rounds of iterative cognitive interviews (CI) with IPV survivors in Iceland where we explored the phenomenon of interest (i.e., help-seeking barriers) qualitatively, pretested and adapted the existing items, and developed new (emic) items based on the survivors’ experiences. Using this data, we utilized building to adapt the BHS-TR scale with the additional emic items and then used psychometric testing in the Quan phase to evaluate the whole instrument with an Icelandic sample of IPV survivors.

### 2.2. Sampling and Data Collection

We used a sequential mixed-methods sampling design with parallel samples [43] for the Qual and Quan phases of the study. The inclusion criteria for both phases were to self-identify as a woman, be 18 or older, live in Iceland, speak Icelandic, and have experienced IPV. In addition, for ethical reasons and the women’s safety, they had to have been out of the abusive relationship for at least a year. Following its aim, a stricter criterion for the participants’ Icelandic language proficiency was applied in the Qual phase; still, to answer the survey, the participants needed to be able to read and understand Icelandic.

This study is part of ongoing international research on help-seeking and trauma recovery carried out by the Multicultural Study of Trauma Recovery (MiStory). The MiStory network (https://mistory-traumarecovery.org/, accessed on 21 November 2021) is a research collaborative currently working in 15 countries around the world to better understand and use safe and trauma-informed methods that illuminate the interactions among cultural context, the self, gender, and recovery.

#### 2.2.1. Qualitative Phase

Three rounds of in-person CI with 17 Icelandic IPV survivors were conducted. The interview procedures were guided by standard CI recommendations [44] and cross-cultural cognitive interviewing [45]. Participants were recruited from centers and service providers for survivors of violence in North and South Iceland. Purposive sampling attempts were made to select diverse individuals within the target population. The years in the abusive relationship ranged from 1 to 18, and most of the women had been in the relationship for over 6 years. The years out of the relationship at the time of the interviews varied from 2 to 12, on average, 5 years. For further information on participants’ characteristics, see Table 1.

The first two rounds consisted of 17 interviews between August and October 2019 and were part of the cross-cultural adaptation and initial testing of the BHT-TR [35]. Based on these interviews, we developed new items for the Icelandic version of the scale and pretested these items in the third round between December 2019 and January 2020. The final round, which was part of member checking and pretesting the new items, involved a second interview with eight of the 17 women who participated in the earlier rounds. The eight women were chosen purposively based on their help-seeking history and experiences with barriers. The Qual data collection continued until data saturation was achieved. All 25 interviews took place in a location chosen by participants and were conducted by the first author. A think-aloud technique and concurrent verbal probing were used to elicit the women’s interpretive process, and the interviews were all audio-recorded with permission.

#### 2.2.2. Quantitative Phase

The Quan phase was advertised using social media posts and flyers posted on various sites, such as centers and services for violence survivors in Iceland, located all over the country. Participants accessed an online survey by following a link hosted on the Icelandic Directorate of Equality’s website. The survey was built using Qualtrics, a secure online platform, and included the measure under study, demographic questions and several other instruments measuring mental and physical health symptoms, help-seeking history, and trauma-recovery related outcomes. A total of 168 women in Iceland who self-identified as IPV survivors answered the survey anonymously collected between February and October 2020. However, the data presented here include only the 137 participants who completed the entire BHS-TR scale (see Table 1).

### 2.3. Ethical Considerations

Permission was obtained from the National Bioethics Committee in Iceland (Qual phase: VSNb2019060009/03.01; Quan phase: VSNb2019090016/03.01), and the study was reported to the Data Protection Authority (Qual phase: 19–119; Quan phase: 19–166). All participants received detailed information about the study and gave their voluntary informed consent before participation. Written consent was obtained from the interview participants, while the survey participants provided their consent by answering the survey. At the end of the interview or the survey, all participants received a list of local referral resources. They were also offered support from a psychologist if difficult emotional reactions emerged during or after participation.

### 2.4. Measures

#### 2.4.1. The Barriers to Help-Seeking for Trauma (BHS-TR) Scale

The BHS-TR asks about barriers to seeking help for trauma recovery in the past year. The instrument’s directions are: “Think about your experiences and feelings that are a result of gender-based violence. In the last 12 months, how much of the feelings or attitudes listed below influenced your decisions not to seek help?” Respondents answer on a 4-point Likert scale anchored at 1 (“Did not influence me”) to 4 (“Strongly influenced me”), with a higher total score indicating more help-seeking barriers [26]. The English BHS-TR consists of seven subscales that can be grouped along two indices. The “Structural Index” includes subscales that represent perceived structural barriers, including the “Unavailable/Not Helpful”, “Financial Concerns”, “Discrimination” and “External Constraints” subscales. The “Internal Index” includes subscales that represent perceived internal barriers and is comprised of the “Shame”, “Frozen/Confused” and “Problem Management Beliefs” subscales. The entire scale was psychometrically sound in a sample of American GBV survivors [34]. The initial CI testing of the Icelandic BHS-TR provided evidence of content validity [35], but further psychometric testing was needed which was a primary aim of this study.

#### 2.4.2. Patient Health Questionnaire

The eight-item version of the Patient Health Questionnaire (PHQ-8) [46] is a valid diagnostic tool that measures depressive symptoms in the general population. It consists of eight of the nine criteria on which the DSM-IV diagnosis of depression is based, omitting the last item on PHQ-9 about suicidal or self-injurious thoughts, making it suitable for more general survey use. Respondents are asked to assess the frequency of symptoms in the past two weeks on a 4-point response scale from 0 (“Not at all”) to 3 (“Nearly every day”), resulting in a total score range from 0 to 24. A clinical cut-off score of ≥10 has been recommended to indicate probable depression [46,47]. The Icelandic version of the measure (or the PHQ-9) has been shown to have sound psychometric properties [48]. Cronbach’s alpha in our sample was 0.87.

#### 2.4.3. Post-Traumatic Stress Disorder (PTSD) Checklist for DSM-5

The PTSD Checklist for DSM-5 (PCL-5) [49] is a widely used and validated measure that assesses the presence and severity of PTSD symptoms. The measure consists of 20 items that correspond with the DSM-5 criteria, and it can be used to screen for probable PTSD. Respondents are asked to rate how bothered they have been by the symptoms in the past month on a 5-point response scale from 0 (“Not at all”) to 4 (“Extremely”), resulting in a total score range from 0 to 80. A clinical cut-off score of ≥31 has been recommended to indicate probable PTSD [49,50,51]. Validation studies on the Icelandic version of the PCL-5 have not been published, but the measure is used in research and clinical work in Iceland. It has been shown to have good internal consistency [52]. Cronbach’s alpha in our sample was 0.96.

#### 2.4.4. Beliefs toward Mental Illness Scale

Beliefs Toward Mental Illness Scale (BTMI) [53] is a mental illness stigma measure that consists of 21 items assessing negative stereotypical views of psychological disorders, including subscales for dangerousness, social dysfunction, incurability, and embarrassment. The scale was designed to measure differences in such views and to predict treatment-seeking behavior among different cultural groups. Participants rate their level of agreement with the belief statements on a 6-point Likert scale ranging from 0 (“Completely disagree”) to 5 (“Completely agree”). Higher scores reflect more stigma towards mental illness. The BTMI has been demonstrated to be reliable and valid across cultures [53,54,55], but validation studies on the Icelandic version have not been published. Cronbach’s alpha for the full scale in our sample was 0.89, with the alpha values for the subscales ranging from 0.71 to 0.81.

#### 2.4.5. Orientation to Life Questionnaire

The shortened version of the Orientation to Life Questionnaire (SOC-13) [56] is a widely used measure that assesses sense of coherence, a concept at the heart of the salutogenic model of health and argued to be an important determinant of successful coping with stressful life situations [57,58,59]. SOC-13 consists of 13 items about how people view their life, measuring the three main components of sense of coherence: comprehensibility, manageability, and meaningfulness. Participants rate their level of agreement or disagreement on a 7-point semantic differential scale, with two anchoring responses adjusted to each item. The total score range is from 13 to 91, and a higher score indicates a stronger sense of coherence. The measure has been found to be reliable and valid in multiple studies conducted in numerous countries [60,61], but validation studies on the Icelandic version of the SOC-13 have not been published. The original full 29-item version in Icelandic has been shown to have good internal consistency reliability [62], and Cronbach’s alpha for SOC-13 in our sample was 0.85.

### 2.5. Data Analysis

#### 2.5.1. Qualitative Phase

Qualitative content analysis (QCA) was chosen to analyze the Qual data as it represents a systematic way to examine both manifest and latent content with the aim of describing a phenomenon and its meaning [63,64].

##### Item Generation

The first 17 interviews had already been transcribed verbatim in former steps of the cross-cultural adaptation process [35]. The domain for the item generation was any barriers to help-seeking after trauma that were not already included in the scale. We used the same framework that guided the development of the BHS-TR, where help-seeking is defined as “the experiences, expectations, and interpretations that interact with structural variables, as well as context, to influence behavior aimed at reducing suffering and promoting health [65] (p. 163)”.

The unit of analysis was the parts of the interviews where the women spoke about help-seeking barriers they thought were missing from the instrument. The data were analyzed using inductive QCA as described by Elo and Kyngäs [63], including making sense of the data to gain a comprehensive understanding of the content, open coding, creating categories, and abstraction. The steps of the process are described in detail in Table 2. After forming and defining the categories, a new item was developed based on each of the sub-categories. Icelandic and English versions of the items were developed simultaneously; however, only the Icelandic ones were tested in this study.

##### Pretesting the New Items

The second interview data were analyzed using deductive QCA as described by Elo and Kyngäs [63]. After each interview, the audio recordings were reviewed, transcribed, and detailed summaries were prepared. These interview summaries were used as the primary source for the analysis, and the data were coded using an unconstrained categorization matrix based on four categories identified in our previous work [35], focusing on the general design, language and wording, cultural relevance, and trauma sensitivity of the items.

In both strands of the Qual analysis, the first author was the primary analyst, responsible for performing each step, with a thorough follow-up and dialogue on the whole process from the other authors. All authors were involved in the careful determination of finalizing the new items, and an audit trail was maintained for scientific rigor.

#### 2.5.2. Quantitative Phase

We began by examining the survey data to ascertain that the variables’ distribution and relationships did not violate the statistical assumptions of the analyses. There were no missing data for the BHS-TR scale as our criteria preclude it, and missing data did not exceed 5% on any of the other measures. All statistical analyses were performed using the SPSS Statistics software package (Version 27.0; IBM Corp., Armonk, NY, USA, 2020), and the significance level was set at *p* ≤ 0.05.

##### Factor Structure and Dimensionality

For item reduction purposes and to explore the underlying factor structure of the 41-item (34 etic and 7 emic) version of the BHS-TR, we conducted a principal component analysis (PCA) following best practices in exploratory factor analysis (EFA) [66,67,68]. Although PCA is not technically a factor analysis method, it is a psychometrically sound and widely used technique for data extraction [66]. To be consistent with the terminology used for the source version of the BHS-TR, we use the term factor instead of component. To determine if the data were suitable for PCA, the correlation matrix was inspected for any variables that had either many items with low (*r*  < 0.30) or high (*r* >  0.90) correlations. In addition, Bartlett’s Test of Sphericity was performed. Furthermore, the Kaiser–Meyer–Olkin (KMO) measure of sampling adequacy was calculated to test whether the sample size was adequate (cut-off > 0.60).

PCA was performed using oblique direct oblimin rotation to allow for the expected correlations between factors and what is recommended when measuring constructs related to human behavior [67]. We retained factors based on three criteria: (1) examination of the scree plot [69], (2) the eigenvalues were greater than 1.0 [70], and (3) the extracted factor solution was deemed interpretable and theoretically sensible [71]. Items were included in a factor if: (1) loading value > 0.35, (2) no cross-loading, and (3) communality value > 0.40 [67,68]. To further explore the measure’s structure, we used multidimensional scaling (MDS) to test if the retained factors would group into the two indices of Structural and Internal Barriers.

##### Convergent and Discriminant Validity

To assess the convergent and discriminant validity of the scale, we examined the associations between help-seeking barriers (using the BHS-TR subscales and indices) and mental illness stigma beliefs (measured with the BTMI and its subscales) on the one hand and sense of coherence (measured with the SOC-13) on the other. For the convergent validity, we hypothesized that barriers to help-seeking, especially the internal barriers, would be related to stigma beliefs and expected there to be moderate to high positive correlations. For the discriminant validity, we hypothesized that barriers to help-seeking would not be highly related to a sense of coherence and expected there to be no or weak negative correlations. These relationships have been indicated in the relevant literature (e.g., [27,57,59,65]) and shown in a prior evaluation of the BHS-TR [34]. We used Cohen’s [72] suggestions for correlation coefficients cut-off values of >0.10 = weak; >0.30 = moderate; >0.50 = strong.

##### Known-Groups Validity

We used independent sample *t*-tests to examine whether the BHS-TR could differentiate between groups known to differ on help-seeking barriers. We created “probable depression” and “probable PTSD” subgroups by using the PHQ-8 clinical cut-off score of ≥10 and the PCL-5 clinical cut-off score of ≥31. Based on the relevant literature (e.g., [24,25,26]) and results from a prior study on the BHS-TR [34], it was expected that survivors with depression or PTSD would on average score higher on the scale than survivors without depression or PTSD.

##### Reliability

The internal consistency of the BHS-TR, its subscales, and indices was assessed using Cronbach’s alpha coefficient. As recommended [73,74], values above 0.70 but not higher than 0.90 were preferred.

## 3. Results

### 3.1. Qualitative Phase

**Figure 1:**
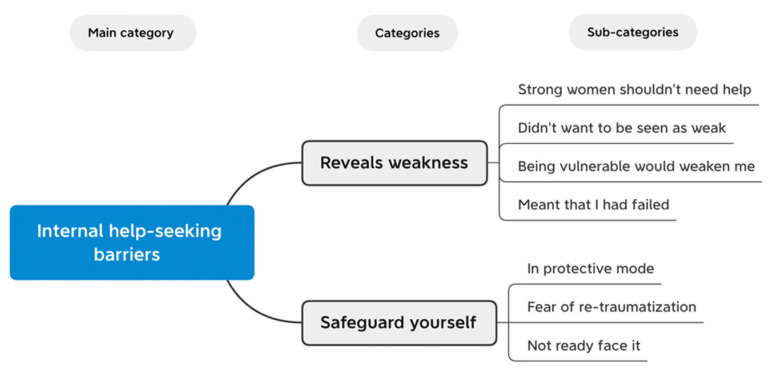
Conceptual diagram of the newly identified help-seeking barriers.

Figure 1 shows the conceptual diagram of the help-seeking barriers identified from the interviews. These barriers were all internal barriers and were conceptually divided into two categories. The first category was “Reveals Weakness”, composed of four sub-categories, and the second category was “Safeguard Yourself”, composed of three sub-categories. The proposed new items developed based on each sub-category are presented in Table 3 and Table 4. Quotations from the women that best represent and support each identified barrier were chosen.

#### 3.1.1. Reveals Weakness

A common thread across all the interviews was beliefs related to viewing help-seeking as a sign of weakness and how it had hindered the women in their help-seeking journey. The Reveals Weakness category (see Table 3) included sub-categories reflecting aspects of this possible factor of help-seeking barriers: (1) strong women should not need help, (2) did not want to be seen as weak, (3) being vulnerable would weaken me, and (4) meant that I had failed.

#### 3.1.2. Safeguard Yourself

The coping of wanting to protect yourself but getting stuck there was commonly mentioned in the interviews as a barrier to help-seeking. The Safeguard Yourself category (see Table 4) included sub-categories reflecting aspects of this possible factor of help-seeking barriers: (1) in protective mode, (2) fear of re-traumatization, and (3) not ready to face it.

#### 3.1.3. Pretesting the New Items

The new items were well-received by the women, who confirmed that the items accurately described their experiences. No issues regarding the general design, relevance, or lack of trauma sensitivity were identified. A few women mentioned minor changes to the wording of some items for clarity, which we changed according to their suggestions before finalizing the items for further evaluation in the Quan phase.

### 3.2. Quantitative Phase

#### 3.2.1. Participants’ Characteristics, Health Status, and Help-Seeking

A total of 137 Icelandic women with a history of IPV participated in the Quan phase (see Table 1). Their ages ranged from 19 to 76 years (M = 40.7, SD = 11.7). Most of the women had junior college or university degrees (81.1%), were employed or students (83.2%), and were mothers (76.7%). The majority self-reported a current mental or physical diagnosis (67.9%). Using the clinical cut-off scores, 41.6% of the participants had probable depression, and 45.3% had probable PTSD. Most of the women (81.8%) had received mental healthcare at some point in their life. When asked about needing help in the last 12 months because of their trauma, 75.9% reported a perceived need for help in general, and 70.1% reported specifically needing mental health treatment. However, almost half of the participants (45.3%) said that they did not seek the professional help they believed they needed in the last 12 months.

#### 3.2.2. Construct Validity

##### Factor Structure

In the initial runs of PCA, following the procedure and criteria described above, we eliminated 15 items that had loading values below 0.35, cross-loadings, or communality values below 0.40. The items that were dropped from the scale are listed in Table 5. Of these items, nine were from the original mental healthcare utilization scale, three were from the trauma-specific additions, and three were from the emic additions we created for this study. After eliminating the problematic items, mainly due to cross-loadings, the final PCA for the remaining 26 items showed that all those items met our criteria. Examination of the correlation matrix from the final run indicated a patterned relationship amongst the variables, and no multicollinearity issues were identified. Bartlett’s test of sphericity (χ^2^(325) = 1705.503, *p* < 0.001) further indicated the patterned relationships and that the correlations between items were sufficient for PCA as well as the use of an oblique rotation. Moreover, the KMO value was 0.79, supporting the sampling adequacy for the analysis.

Eight factors had eigenvalues greater than 1.0. but the scree plot was somewhat ambiguous, showing inflections that would justify retaining from six to eight factors. Since a seven or eight-factor solution was deemed theoretically sensible and was consistent with the factor structure of the English BHS-TR scale, but still allowing for an additional emic factor, we retained eight factors. These factors combined explained 72.14% of the total variance, and the factor loadings after rotation (pattern matrix) are shown in Table 6. The items that clustered together suggest that factor 1 represents “Weakness/Vulnerability” barriers, factor 2 represents “Financial Concerns” barriers, factor 3 represents “Unavailable/Not Helpful” barriers, factor 4 represents “External Constraints” barriers, factor 5 represents “Problem Management Beliefs” barriers, factor 6 represents “Frozen/Confused” barriers, factor 7 represents “Inconvenience” barriers, and factor 8 represents “Shame” barriers. The following analyses further assessed this initial eight-factor solution of the Icelandic BHS-TR, and the factors were labeled and evaluated as potential subscales.

##### Structural and Internal Barriers Indices

**Figure 2:**
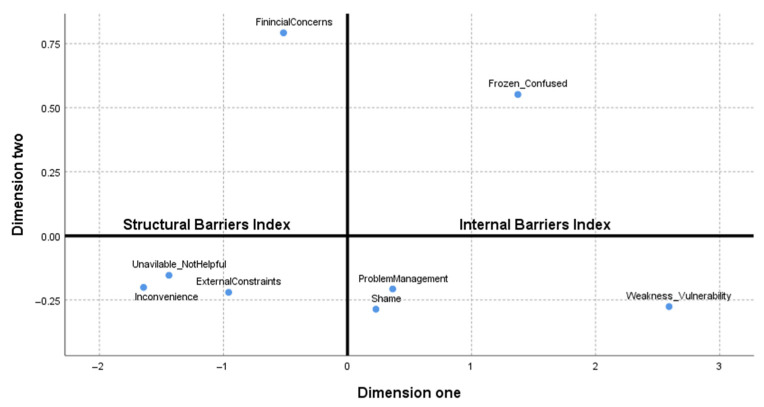
The structural and internal barriers indices of BHS-TR.

The results from the MDS showed that Financial Concerns, Unavailable/Not Helpful, External Constraints, and Inconvenience were a dimension of barriers that, when grouped, comprised the “Structural Barriers Index”. Weakness/Vulnerability, Problem Management Beliefs, Frozen/Confused and Shame barriers were a separate dimension, and when grouped, comprised the “Internal Barriers Index” (see Figure 2). We computed these two indices to use along with specific barrier subscales sum scores for further validation analyses.

##### Convergent and Discriminant Validity

There were no significant correlations between the scores on the BTMI scale and the scores on the Structural or Internal Barriers indices. However, there were weak and moderate significant positive correlations between the scores on the Embarrassment BTMI subscale and the scores for the BHS-TR subscales of Problem Management Beliefs (*r* = 0.22, *p* = 0.02), Shame (*r* = 0.38, *p* = 0.00), and Weakness/Vulnerability (*r* = 0.41, *p* = 0.00).

There was no significant correlation between the scores on the SOC-13 scale and the scores on the Structural Barriers Index. However, there was a weak significant negative correlation with the scores on the Internal Barriers Index (*r* = −0.21, *p* = 0.02). The SOC-13 scores were unrelated to most of the BHS-TR subscales. Still, there were weak significant negative correlations for SOC-13 and the Unavailable/Not Helpful (*r* = −0.17, *p* = 0.04), Weakness/Vulnerability (*r* = −0.18, *p* = 0.04), and Frozen/Confused (*r* = −0.22, *p* = 0.01) subscales.

##### Known-Groups Validity

The total mean score of the BHS-TR scale and mean scores of the Structural and Internal Barriers indices were all significantly higher for the probable depression and probable PTSD groups (see Table 7). In addition, most of the subscales’ mean scores were significantly higher for the probable depression and probable PTSD groups. However, there were no significant differences in the mean scores for the groups for the Financial Concerns, Weakness/Vulnerability, and Problem Management Beliefs barriers. Furthermore, the mean score for the Unavailable/Not Helpful subscale was significantly higher only for the depression group, and the mean score for the Shame subscale was significantly higher only for the PTSD group.

#### 3.2.3. Reliability

The 26-item scale showed good internal consistency with Cronbach’s alpha of 0.87, and the values for the Structural and Internal Barriers indices were 0.75 and 0.88, respectively. Moreover, all except two of the subscales had Cronbach’s alpha exceeding 0.70. By examining the Cronbach’s alpha if an item was deleted, all 26 items appeared worthy of retention. Therefore, no items were dropped based on the reliability analysis, and the items in the subscales with alpha values below 0.70 were retained for continuing research and further evaluation. Descriptive results and alpha values for the full scale are presented in Table 8.

## 4. Discussion

This study described the development and psychometric evaluation of the Icelandic version of the BHS-TR, the first trauma-specific and survivor-centered measure on help-seeking barriers available in Iceland. The mixed-methods approach provided a rigorous process, capitalizing on the strengths of both Qual and Quan methods while minimizing the weaknesses [40,42]. The use of mixed methods in instrument development and validation studies has been increasing. It can promote more rigor in the process, optimizing the development of psychometrically sound and culturally sensitive instruments [75,76,77]. The combined etic–emic strategy, allowing for the development and adding of the new items, was also valuable to enhance the Icelandic BHS-TR’s construct validity and cultural sensitivity. This approach has been successfully used in other studies adapting measures developed in other countries to the Icelandic context [78,79].

It is noteworthy that while the new items are labeled as emic (culture-specific) because they were developed based on Icelandic survivors’ experiences, we recognize that these help-seeking barriers are most likely not specific to Iceland. Fearing vulnerability and not wanting to appear weak to others is a significant challenge for trauma recovery amongst GBV survivors in other countries [23,80]. Moreover, barriers similar to our safeguard barriers were identified in a recent systematic review on mental health service use among trauma survivors, where concerns about re-experiencing the traumatic events and avoiding reminders were prominent [81]. Nonetheless, it remains an empirical question whether any of the new items have value for use in other cultures. Our research group is currently testing these items as possible additions for the English BHS-TR in American samples.

The examination of the Icelandic BHS-TR structure was exploratory following the emic approach, as ‘imposed etics’ or, in this case, a measure’s structure, may obscure culture-specific results [38,82]. Our study revealed eight underlying factors (possible subscales) of the BHS-TR. This process reduced the number of items to 26, and 20 of those are shared with the 24-item English version of the scale [34]. Six of the eight factors (Financial Concerns, Unavailable/Not Helpful, External Constraints, Problem Management Beliefs, Frozen/Confused, and Shame) were generally identical to the seven-factor model of the English BHS-TR. Moreover, further supporting the construct validity of the measure, our results also showed two indices of Structural and Internal Barriers.

Interestingly, the Reveals Weakness category became an emic factor but not the Safeguard Yourself category. However, besides the three weakness items, this new Weakness/Vulnerability factor included one safeguard item (“Seeking help would require acknowledging things I did not want to face”), and one privacy item (“I thought my situation was too personal or wanted to keep it private”) that belonged to the Shame subscale on the English BHS-TR. Surprisingly, the weakness item that got dropped in the Quan phase was a vulnerability barrier (“I felt like opening up to my feelings would weaken me”), which was central in the Qual phase’s survivors’ narratives. Other items that were surprisingly dropped were the trauma-specific additions about the notion that other people would not understand or could not be trusted to help (see Table 5), despite the fact that perceived rejection and mistrust of people or systems are commonly cited barriers in previous studies [20,26,83]. Most of these items were dropped due to cross-loadings on different factors. While we know this concept is critical based on our Qual work, we may need to revise the wording of the items to capture the concept more fully. Additional study is underway to critically evaluate these items.

Another result from the Quan phase raising concerns was that the entire Discrimination factor from the English BHS-TR, including items about culture, background, and prejudice, was dropped. The relevance of these items had been questioned in the CI pretesting [35], and it is possible that this subscale performed poorly in our study because of the ethnic and socio-economic homogeneity of our Icelandic sample. Future studies can examine this by carrying out sampling of people with more diverse backgrounds and immigrant status.

Finally, the structural items about distance or transportation problems and seeking help taking too much time hung together as a separate factor, which is not consistent with the English BHS-TR structure. We chose to include this possible Inconvenience subscale for the Icelandic BHS-TR and continue the research on this concept, as these barriers might be important in the Icelandic help-seeking context.

The full 26-item BHS-TR, the two indices, and most subscales showed good reliability as measured with Cronbach’s alpha coefficient. The two subscales (Problem Management Beliefs and Inconvenience) with alpha values below the recommended 0.70 are short subscales with only three and two items, respectively. Cronbach’s alpha is sensitive to the number of items. It is expected to find relatively low values with short scales, and some researchers claim that the coefficient is inappropriate and even meaningless for two-item scales [84,85]. Still, these results further indicated the need to continue working with the subscales, especially the inconvenience one, as stable factors usually need to include at least three items [67,68].

Our results demonstrate support for the Icelandic BHS-TR convergent and discriminant validity by showing the hypothesized relationships between help-seeking barriers and mental illness stigma beliefs on the one hand and sense of coherence on the other. Nonetheless, the correlations between the barriers and stigma beliefs were only weak to moderate. In addition, the results provided evidence of known-group validity, as the BHS-TR could differentiate between groups of IPV survivors based on the severity of depression and PTSD symptoms.

Several limitations of this study should be noted, including relatively small, self-selected samples of IPV survivors in both phases. As with most other studies among hidden populations [86], probability sampling was impossible. Using an online survey for the Quan phase also limited our ability to reach individuals without access to a computer or with limited technological literacy. While a larger sample, especially for examining the BHS-TR structure, would have been preferred, testing the sampling adequacy showed that our sample size was sufficient. Furthermore, previous studies have revealed that EFA and PCA can be applied to sample sizes far below what traditional recommendations suggest and still yield reliable results [87,88].

Another limitation is that for the Quan sample, we did not have information on how long the women had been in the abusive relationship, the time out of it, their race, or foreign origin. Both the time in and out of the abusive relationship varied for the women in the Qual sample, but they were predominantly Caucasian and born in Iceland. Since the participants needed to read and understand Icelandic to take the survey, those characteristics might also be the case for the Quan sample, which could explain why the Discrimination subscale did not remain a factor in our study. This is noteworthy because women of foreign origin are increasingly making Iceland their home and it is well-established that immigrants may be particularly at risk for violence in intimate relationships and might have even more difficulties seeking help than their Icelandic-born counterparts [16,89]. Although participants in both of our samples had experienced many barriers, most of these women had at some point sought help and care. Considering the literature reviewed above, demonstrating that many survivors never seek help, even despite the perceived need, the scale must capture the barriers experienced by an increasingly diverse group of survivors.

Future work with and about the BHS-TR would be greatly enhanced in studies with more extensive and more diverse samples regarding, e.g., gender, foreign origin, types of GBV experienced, disabilities, and help-seeking history. While the exploratory nature of this study yielded important findings, future research should include the use of confirmatory factor analysis and test-retest reliability assessment. Finally, while the strengths of the etic–emic strategy have been pointed out, using this approach can limit the equivalence between language versions of a measure, thus making cross-cultural comparisons challenging. One recommendation to address this issue is to use only shared (etic) items for comparison [38]. As has been noted, we are currently testing the newly developed BHS-TR items for use outside of Iceland.

## 5. Conclusions

Taken together, the results of this study indicate that the Icelandic BHS-TR is reliable, valid, and helpful in understanding aspects of the help-seeking barriers that IPV survivors in Iceland face. The 26-item version with eight subscales and two indices shows promise but deserves continuing attention for improvement and maximum utility to populations not well-represented in our samples.

The availability of this psychometrically sound and survivor-centered measure on help-seeking barriers has value by providing information that can guide the development of evidence-based interventions targeted toward breaking down barriers and increasing help-seeking among survivors. Understanding these barriers can also guide trauma-informed practice that integrates a better understanding of trauma responses and sociocultural influencing factors into serveries where survivors might seek help in their journey to trauma recovery.

## Figures and Tables

**Table 1:** Overview of the participants’ demographics characteristics.

Characteristics	Qual Phase (*n* = 17)	Quan Phase (*n* = 137)
**Age**		
18–29	4 (23.5%)	24 (17.5%)
30–39	7 (41.2%)	34 (24.8%)
40–49	4 (23.5%)	38 (27.7%)
50–59	1 (5.9%)	18 (13.1%)
60+	1 (5.9%)	6 (4.4%)
Not stated	–	17 (12.4%)
**Racial and ethnic background**		
Caucasian	17 (100%)	–
Iceland-born	16 (94.1%)	–
Foreign-born	1 (5.9%)	–
**Level of education**		
High school or less	3 (17.6%)	11 (8.0%)
Technical or junior college degree	5 (29.4%)	29 (21.2%)
University degree	9 (52.9%)	82 (59.9%)
Not stated	–	15 (10.9%)
**Employment status** (not mutually exclusive)		
Working	12 (70.6%)	88 (64.2%)
Unemployed or looking for work	2 (11.8%)	7 (5.1%)
Student	5 (29.4%)	26 (19.0%)
Homemaker	1 (5.9%)	3 (2.2%)
Unable to work due to sickness/disability	3 (17.6%)	20 (14.6%)
Other	–	24 (17.5%)
**Number of children**		
None	5 (29.4%)	24 (17.5%)
One or two	9 (52.9%)	59 (43.1%)
Three or more	3 (17.6%)	46 (33.6%)
Not stated	–	8 (5.8%)
**Current medical diagnosis (mental and/or physical)**		
No	6 (35.3%)	44 (32.1%)
Yes	11 (64.7%)	93 (67.9%)
**History of receiving mental healthcare**		
No	8 (47.1%)	24 (17.5%)
Yes	9 (52.9%)	112 (81.8%)
Not stated	–	1 (0.7%)

**Table 2:** Overview of the content analysis process, based on Elo and Kyngäs [63].

QCA Inductive Approach	Description of How Each Step Was Performed
Preparation phase	
Selecting the unit of analysis	In accordance with the aim of the study, it was decided to analyze the parts of the interviews where the women spoke about help-seeking barriers they thought were missing from the scale.
Making sense of the data and obtaining a whole	First, the transcribed material was read through several times without coding to become immersed in the data.
Organizing phase	
Open coding	The transcribed material was read through again, and this time paragraphs and phrases (meaning units) directly related to the phenomenon under study were highlighted, and headings written down in the margins. Next, the headings were collected onto coding sheets and categories freely generated.
Grouping and categorization	The initial categories were compared and grouped based on similarities and differences into broader higher-order categories.
Abstraction	Each category was defined and named according to its content. Sub-categories with similar meanings were then grouped to form categories, which were all classified under the main category. The analysis process was continued until new categories could no longer be formed.
Reporting phase	
Report on the process	Each step was thoroughly documented during the process, allowing tracking of all the decisions made. This article represents the final step, where the analysis and findings are reported.

**Table 3:** Reveals weakness category.

Barriers Reveals Weakness	Meaning Units	Proposed New Items
Strong women shouldn’t need help	“It was so strong within me the need to be tough and keep going, needing help felt like a sign of weakness” “I wanted to feel strong, show some strength, we are supposed to be so hardy and resilient…you know Vikings or whatever, and I guess that some part of me believed that strong women shouldn’t need help”	I thought that strong people should not need help
Didn’t want to be seen as weak	“I didn’t want to be looked at as weak and then to have people treat me differently…you know, feel sorry for me” “I was scared of being seen as a weakling”	I was scared of being seen as weak
Being vulnerable would weaken me	“It was like I would somehow become less…I don’t like being vulnerable, and vulnerability is to me at least a big part of seeking help” “I felt like, if I would open up…you know about my feelings or whatever that it would weaken me”	I felt like opening up to my feelings would weaken me
Meant that I had failed	“It was like a defeat or something, like such a personal failure, and that’s why it took me such a long time to seek help, it wasn’t until I had nothing left” “To seek help would mean that I had ultimately lost, for him and what he did to be the reason I was so fucked up…and like still…I couldn’t bear it”	Getting help would mean that I had failed or had been defeated

**Table 4:** Safeguard Yourself category.

Barriers Safeguard Yourself	Meaning Units	Proposed New Items
In protective mode	“I wanted to protect myself, and I was in this mode that I just could not deal with it and needed to let myself be there…but you shouldn’t stay there for too long, you can get stuck” “I didn’t want to take the chance of regretting it. You know if I would seek help, and I wouldn’t be believed, or it wouldn’t be taken seriously, I was dealing with enough”	I did not seek help in an effort to protect or safeguard myself
Fear of re-traumatization	“I had made my world trigger-free, so yeah, I was really isolated but it was easier that way, and I just didn’t see the point…to go there, talking about it would only hurt me even more” “I was afraid that it would be too difficult for me because then I had to think about it, talk about it, recall these painful memories, and there was no way that I could do that”	I was afraid that seeking help would be too emotionally difficult or hurt me even more
Not ready to face it	“The desire to be whole was so strong, and if I had to get help, that would mean that I wasn’t whole anymore…of course, deep down, I knew I was broken, but I wasn’t ready to admit it” “Denial was a huge barrier for me, because you know, staying in denial doesn’t hurt as much…if you seek help, you need to face your experience”	Seeking help would require acknowledging things I did not want to face

**Table 5:** Items removed from the Barriers to Help-Seeking for Trauma (BHS-TR) scale in the Quan phase.

Dropped Items
From the original mental healthcare scale
3. I was unsure about where to go for help or how to access help
4. I thought help probably would not do any good
9. I could not get time away from work or my family
12. I was concerned that I would not be able to get help soon enough
13. I was scared about being put into a hospital against my will
20. I felt that my culture, background, or specific situation would not be understood
21. Suitable professionals were not available to me
22. The kind of help I needed was not available
23. I felt that there would be prejudice or discrimination against me
From the trauma-specific additions
31. I was afraid I would explain what I needed, and no one would help me anyway
32. I felt that I could not trust people to help me
33. I felt no one could understand or help me
From the cognitive interviews (emic) additions
36. I was afraid that seeking help would be too emotionally difficult or hurt me even more
37. I did not seek help in an effort to protect or safeguard myself
38. I felt like opening up to my feelings would weaken me

**Table 6:** The eight-factor solution of the Icelandic BHS-TR scale.

Factors Items (Communalities)	1	2	3	4	5	6	7	8
Weakness/Vulnerability (Cumulative % of Variance: 26.47; Eigenvalue: 6.88)								
40. I thought that strong people should not need help (0.84)	**0.93**							
39. Getting help would mean that I had failed or had been defeated (0.81)	**0.83**							
35. I was scared of being seen as weak (0.79)	**0.72**				0.22			
41. Seeking help would require acknowledging things I did not want to face (0.68)	**0.69**					0.34		
24. I thought my situation was too personal or wanted to keep it private (0.50)	**0.40**		0.27					0.27
Financial Concerns (Cumulative % of Variance: 37.96; Eigenvalue: 2.99)								
2. I was concerned that the help I needed would be too expensive (0.80)		**0.87**						
19. The available health insurance would not cover the type of treatment I needed (0.78)		**0.85**					0.28	
18. I did not have adequate financial resources (0.81)		**0.82**						
Unavailable/Not Helpful (Cumulative % of Variance: 45.98; Eigenvalue: 2.07)								
15. I was not satisfied with the available services (0.75)			**0.87**					
16. I felt that the help available would not provide the type of treatment or help that was best for the problem (0.72)			**0.85**					
17. I had sought help before, but it did not help (0.60)			**0.61**	0.32		−0.24		
External Constraints (Cumulative % of Variance: 53.06; Eigenvalue: 1.84)								
14. I was worried that if others discovered my health problems or situation, I could lose my children, security, or housing (0.74)				**0.86**				
34. Others were preventing me from getting the help I needed (0.71)				**0.78**				
25. I was afraid of the consequences for myself, my children, or my family (0.71)				**0.72**				0.32
Problem Management Beliefs (Cumulative % of Variance: 58.38; Eigenvalue: 1.38)								
1. I thought the problem would probably get better by itself (0.59)					**0.77**			
11. I thought the situation was normal or was not severe (0.69)	0.21				**0.62**	−0.34		
10. I wanted to or thought I should solve the problems on my own (0.64)					**0.58**			0.31
Frozen/Confused (Cumulative % of Variance: 63.54; Eigenvalue: 1.34)								
29. I could not seem to clarify my feelings or know what I needed (0.83)						**−0.91**		
30. I was afraid I could not clearly express what I needed (0.67)					0.22	**−0.61**		
26. I was confused or unable to plan out all the details or steps (0.69)				0.24	−0.20	**−0.56**		0.31
27. I felt paralyzed or frozen and unable to get started (0.70)		0.26		0.30		**−0.43**		0.25
Inconvenience (Cumulative % of Variance: 68.08; Eigenvalue: 1.18)								
5. I had distance or transportation problems (0.78)							**−0.83**	
8. I thought getting help would take too much time or was inconvenient (0.64)	0.27				0.22		**−0.60**	
Shame (Cumulative % of Variance: 72.14; Eigenvalue: 1.06)								
6. I was concerned about what others might think (0.80)								**0.86**
7. I was ashamed (0.72)								**0.79**
28. I believed that people would judge me (0.75)								**0.75**

*Notes*: Rotation method: oblimin with Kaiser normalization; all loadings below 0.2 were suppressed for readability.

**Table 7:** Known-groups validity results.

BHS-TR	Depression			PTSD		
Indices and Subscales	No (*n* = 80)	Probable (*n* = 57)	*p*	No (*n* = 75)	Probable (*n* = 62)	*p*
*Structural Barriers*	20.4 (5.4)	23.7 (7.7)	0.00	20.5 (5.5)	23.3 (7.5)	0.01
Financial Concerns	7.0 (3.2)	7.3 (3.4)	–	6.9 (3.3)	7.4 (3.2)	–
Unavailable/Not Helpful	4.4 (2.0)	5.5 (2.7)	0.01	4.6 (2.2)	5.1 (2.6)	–
External Constraints	5.2 (2.5)	6.7 (3.2)	0.00	5.2 (2.6)	6.6 (3.1)	0.00
Inconvenience	3.8 (1.8)	4.4 (1.9)	0.05	3.7 (1.7)	4.5 (2.0)	0.02
*Internal Barriers*	39.9 (10.5)	43.8 (9.9)	0.03	39.8 (10.9)	43.6 (9.5)	0.04
Weakness/Vulnerability	12.6 (4.8)	13.9 (4.7)	–	12.6 (4.9)	13.7 (4.7)	–
Problem Management Beliefs	8.7 (2.5)	9.0 (2.4)	–	8.6 (2.7)	9.1 (2.1)	–
Frozen/Confused	10.5 (3.4)	12.3 (3.0)	0.00	10.7 (3.6)	12.0 (3.2)	0.03
Shame	8.0 (2.9)	8.6 (3.0)	–	7.8 (3.0)	8.8 (2.8)	0.05
Total	60.3 (13.2)	67.5 (15.0)	0.00	60.3 (13.7)	66.9 (14.3)	0.00

*Notes*: Independent sample *t*-tests; mean score (standard deviation); significance level at *p* ≤ 0.05; Patient Health Questionnaire-8 cut-off score of ≥ 10; Post-Traumatic Stress Disorder (PTSD) Checklist for DSM-5 cut-off score of ≥31.

**Table 8:** Descriptive results and alpha values for the BHS-TR scale (*n* = 137).

Indices and Subscales	Min.	Max.	M	SD	α
*Structural Barriers*	11	44	21.8	6.6	0.75
Financial Concerns	3	12	7.1	3.3	0.82
Unavailable/Not Helpful	3	12	4.8	2.4	0.71
External Constraints	3	12	5.8	2.9	0.77
Inconvenience	2	8	4.0	1.8	0.52
*Internal Barriers*	15	60	41.5	10.4	0.88
Weakness/Vulnerability	5	20	13.1	4.8	0.86
Problem Management Beliefs	3	12	8.8	2.5	0.62
Frozen/Confused	4	16	11.2	3.3	0.79
Shame	3	12	8.3	2.9	0.83
Total	26	104	63.3	14.3	0.87

*Notes*: M = mean; SD = standard deviation; α = Cronbach’s alpha.

## Data Availability

The data presented in this study are available on reasonable request from the corresponding author. The data are not publicly available due to ethical and privacy reasons.
